# Glial cell type-specific gene expression in the mouse cerebrum using the *piggyBac* system and in utero electroporation

**DOI:** 10.1038/s41598-021-84210-z

**Published:** 2021-03-01

**Authors:** Toshihide Hamabe-Horiike, Kanji Kawasaki, Masataka Sakashita, Chihiro Ishizu, Tomokazu Yoshizaki, Shin-ichi Harada, Keiko Ogawa-Ochiai, Yohei Shinmyo, Hiroshi Kawasaki

**Affiliations:** 1 Department of Medical Neuroscience, Graduate School of Medical Sciences, Kanazawa University, Takara-machi 13-1, Kanazawa, Ishikawa 920-8640 Japan; 2 Center for Biomedical Research and Education, Graduate School of Medical Sciences, Kanazawa University, Kanazawa, Ishikawa 920-8640 Japan; 3 Department of Otolaryngology-Head and Neck Surgery, Graduate School of Medical Sciences, Kanazawa University, Kanazawa, Ishikawa 920-8640 Japan; 4 Department of Japanese-Traditional (Kampo) Medicine, Kanazawa University Hospital, Ishikawa, 920-8640 Japan

**Keywords:** Developmental neurogenesis, Development of the nervous system

## Abstract

Glial cells such as astrocytes and oligodendrocytes play crucial roles in the central nervous system. To investigate the molecular mechanisms underlying the development and the biological functions of glial cells, simple and rapid techniques for glial cell-specific genetic manipulation in the mouse cerebrum would be valuable. Here we uncovered that the *Gfa2* promoter is suitable for selective gene expression in astrocytes when used with the *piggyBac* system and in utero electroporation. In contrast, the *Blbp* promoter, which has been used to induce astrocyte-specific gene expression in transgenic mice, did not result in astrocyte-specific gene expression. We also identified the *Plp1* and *Mbp* promoters could be used with the *piggyBac* system and in utero electroporation to induce selective gene expression in oligodendrocytes. Furthermore, using our technique, neuron-astrocyte or neuron-oligodendrocyte interactions can be visualized by labeling neurons, astrocytes and oligodendrocytes differentially. Our study provides a fundamental basis for specific transgene expression in astrocytes and/or oligodendrocytes in the mouse cerebrum.

## Introduction

Glial cells such as astrocytes and oligodendrocytes play crucial roles in the central nervous system. Astrocytes perform diverse functions including supplying nutrients to neurons, recycling neurotransmitters and contributing to the blood–brain barrier^1–3^, whereas oligodendrocytes produce myelin to improve the conduction velocity of axons^4–6^. In addition to these classical roles of astrocytes and oligodendrocytes, recent studies have uncovered additional functions. Astrocytes interact with synapses to regulate their formation, maturation and functions^7–9^. Astrocyte-secreted proteins that regulate synaptic formation and elimination have been identified^7,10^. It was shown that oligodendrocytes have the ability to inhibit axonal regeneration^11,12^. Thus, uncovering the biological roles and developmental processes of glial cells has continued to make significant contributions to our understanding of the functions of the brain.

To investigate the molecular mechanisms underlying the development and the biological functions of glial cells, glial cell-specific genetic manipulation is crucial. Currently, transgenic mice and knockin mice using cell type-specific genes have been widely used to manipulate gene expression in astrocytes or oligodendrocytes selectively^13–18^. However, making appropriate transgenic mice and knockin mice is relatively arduous, costly and time-consuming. Therefore, simple and rapid techniques which enable cell type-specific manipulation of gene expression in the brain have been of great interest.

Recently, in utero electroporation has become increasingly popular as a tool to manipulate gene expression in neurons of the cerebral cortex in rodents and carnivores^19–23^. It takes only a few hours to perform in utero electroporation, and in combination with the CRISPR/Cas9 system, not only overexpression of transgenes but also loss-of-function studies can be carried out in cortical neurons^24–26^. Recent studies demonstrated that when using the *piggyBac* system, in utero electroporation can induce transgene expression not only in neurons but also in astrocytes and oligodendrocytes^27–29^. Furthermore, incorporation of glial cell-specific promoters into the *piggyBac* system allowed preferential transgene expression in glial cells in the rodent cerebral cortex^30,31^. Despite these recent advances in transgene expression in glial cells, especially when being used with the *piggyBac* system and in utero electroporation^32^, promoters which are highly specific to particular types of glial cells had not been identified or even investigated carefully. Therefore, here we searched for promoters which are specific to astrocytes or oligodendrocyte in the mouse cerebral cortex when used with the *piggyBac* system and in utero electroporation. We identified a *GFAP* promoter which led to astrocyte-specific transgene expression, and a *Plp1* promoter and an *Mbp* promoter which resulted in oligodendrocyte-specific transgene expression. Importantly, the *Blbp* promoter, which has been used to induce astrocyte-specific gene expression in transgenic mice, did not result in astrocyte-specific gene expression when used with the *piggyBac* system and in utero electroporation. This result indicates that even though some promoters are used to make transgenic mice with specific gene expression patterns, it does not necessarily mean that these promoters also provide the same specific gene expression when used with the *piggyBac* system and in utero electroporation. Furthermore, using our technique, the neuron-astrocyte or neuron-oligodendrocyte interactions can be visualized by labeling neurons, astrocytes and oligodendrocytes differentially. Our findings provide a fundamental basis for specific transgene expression in astrocytes and/or oligodendrocytes in the mouse cerebral cortex.

## Results

### Combining in utero electroporation with the *piggyBac* system leads to transgene expression not only in neurons but also in glial cells in the mouse cerebral cortex

**Figure 1:**
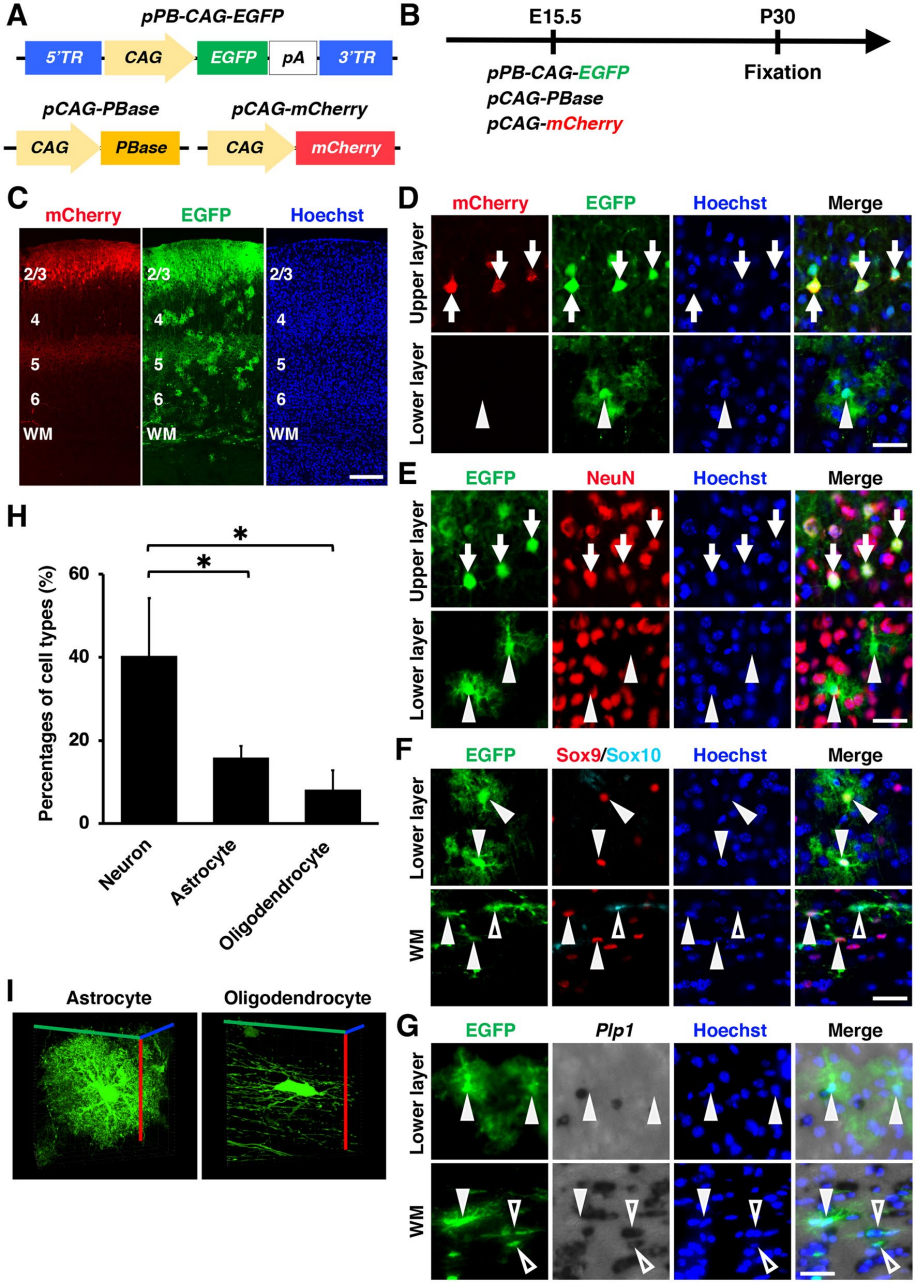
In utero electroporation using the *piggyBac* system led to transgene expression in both neurons and glial cells in the mouse cerebrum. (**A**) Schematics of *pPB-CAG-EGFP*, *pCAG-PBase* and *pCAG-mCherry* plasmids. *pPB-CAG-EGFP* drives EGFP expression under the CAG promoter. *pCAG-PBase* has a *piggyBac* transposase, which recognizes the terminal region (TR) in *piggyBac* donor plasmids. (**B**) Experimental procedure. *piggyBac* plasmids (*pPB-CAG-EGFP* and *pCAG-PBase*) plus *pCAG-mCherry* were co-transfected into the mouse cerebrum using in utero electroporation at E15.5, and coronal sections were prepared at P30. (**C, D**) Coronal sections of the electroporated cerebrum. Higher magnification images corresponding to upper and lower layers of the cerebral cortex in (**C**) are shown in (**D**). In utero electroporation with the *piggyBac* system induced EGFP expression not only in mCherry-positive layer 2/3 neurons (arrows) but also in mCherry-negative cells (arrowheads). (**E–G**) Immunohistochemistry for NeuN (**E**), Sox9 and Sox10 (**F**), and in situ hybridization for *Plp1* (**G**). EGFP-positive cells in upper layers were positive for NeuN (arrows), suggesting that they are neurons. Many EGFP-positive cells in lower layers were positive for Sox9 but negative for NeuN, Sox10 and *Plp1* (arrowheads), suggesting that they are astrocytes. Many EGFP-positive cells in the white matter were positive for Sox10 and *Plp1* (open arrowheads), suggesting that they are oligodendrocytes. (**H**) The percentages of EGFP-positive cells which were also NeuN-positive (neuron), Sox9-positive/Sox10-negative (astrocyte) or *Plp1*-positive (oligodendrocyte). Unpaired Student’s *t*-test, *P < 0.05. Error bars represent mean ± SD. The statistical analyses were performed using Microsoft Excel ver. 16.43. **(I)** Three-dimensional reconstructed images of an EGFP-positive astrocyte (left) and an EGFP-positive oligodendrocyte (right). Scale bars: 200 μm (**C**), 30 μm (**D-G**). WM, white matter. Numbers indicate layers in the cerebral cortex.

We first made the donor plasmid *pPB-CAG-EGFP* using the *piggyBac* system and the CAG promoter (Fig. 1A). We then tested whether the combination of our plasmids using the *piggyBac* system and in utero electroporation results in transgene expression not only in neurons but also in glial cells using the mouse cerebral cortex. We introduced the *piggyBac* donor plasmid *pPB-CAG-EGFP* and the helper plasmid *pCAG-PBase* along with *pCAG-mCherry*, which was used to label transfected cells, into layer 2/3 neurons of the developing mouse cerebral cortex using in utero electroporation at embryonic day 15.5 (E15.5) (Fig. 1A, B). Brain samples were collected at postnatal day 30 (P30), and coronal sections of the cerebral cortex were prepared (Fig. 1B, C). As reported previously^33^, mCherry expression was observed in layer 2/3 cortical neurons (Fig. 1C), indicating that plasmids were introduced into layer 2/3 neurons selectively.

In contrast to mCherry, EGFP, which was expressed using the *piggyBac* system, was expressed not only in mCherry-positive layer 2/3 neurons (Fig. 1D, arrows) but also in mCherry-negative cells both in the gray matter and in the white matter (Fig. 1C, D, arrowheads). Interestingly, the morphology of EGFP-positive/mCherry-negative cells in lower layers of the cerebral cortex seemed like glial cells (Fig. 1D, arrowheads). To investigate the identities of EGFP-positive/mCherry-negative cells, we performed immunohistochemistry. While EGFP-positive cells in upper layers expressed the neuronal marker NeuN (Fig. 1E, arrows), those in lower layers were NeuN-negative (Fig. 1E, arrowheads). Furthermore, many EGFP-positive cells in lower layers were positive for Sox9 but negative for Sox10 and *Plp1* (Fig. 1F, G, arrowheads). These results indicate that EGFP-positive cells in lower layers of the cerebral cortex were mainly astrocytes. Many EGFP-positive cells in the white matter expressed Sox10 and *Plp1* (Fig. 1F, G, open arrowheads), suggesting that EGFP-positive cells in the white matter also contained oligodendrocytes.

We then quantified the percentages of neurons, astrocytes and oligodendrocytes among EGFP-positive cells. 40%, 16% and 8% of EGFP-positive cells were NeuN-positive neurons, Sox9-positive/Sox10-negative astrocytes and *Plp1*-positive oligodendrocytes, respectively (neuron, 40.4 ± 13.8%; astrocyte, 15.9 ± 2.8%; oligodendrocyte, 8.2 ± 4.6%) (Fig. 1H). Consistent with our results, previous studies reported that in utero electroporation using the *piggyBac* system led to efficient gene expression in both neurons and glial cells^27–29^. Thus, these results indicate that the combination of in utero electroporation and the *piggyBac* system can be used to efficiently express transgenes in both neurons and glial cells in the developing mouse cerebral cortex. Furthermore, these findings indicate that *piggyBac* donor plasmids are successfully integrated into the genomic DNA of neural progenitors.

Importantly, higher magnification confocal images revealed clear morphologies of astrocytes and oligodendrocytes in the cerebral cortex (Fig. 1I). The star-shaped, small cell bodies and many fine branches of astrocytes (Fig. 1I, left, Supplementary Movie 1), and the many parallel branches of oligodendrocytes (Fig. 1I, right, Supplementary Movie 2) were clearly visualized. These results indicate that the combination of in utero electroporation and the *piggyBa*c system is useful for investigating the detailed morphological features of astrocytes and oligodendrocytes in the cerebral cortex.

### A promoter suitable for astrocyte-specific gene expression

Although the combination of in utero electroporation and the *piggyBac* system is useful for expressing transgenes in neurons, astrocytes and oligodendrocytes, it would be beneficial if transgenes could be selectively expressed in either astrocytes or oligodendrocytes. To restrict transgene expression patterns, previous pioneering works incorporated several promoters into the *piggyBac* system^30,31^. On the other hand, however, the specificities of promoters used with the *piggyBac* system and in utero electroporation still remained to be investigated.

To find a promoter suitable for glial cell type-specific gene expression using the *piggyBac* system and in utero electroporation, we carefully investigated the transgene expression patterns driven by several promoters. We utilized promoters which were reported to drive selective gene expression in astrocytes using transgenic mice. We used the *Gfa2* promoter, which was derived from the human *glial fibrillary acidic protein* (*GFAP*) gene, and a promoter region derived from the *brain lipid-binding protein* (*Blbp*, also known as *fatty acid binding protein 7*) gene^16,17^ (See Supplementary Table for details).

**Figure 2:**
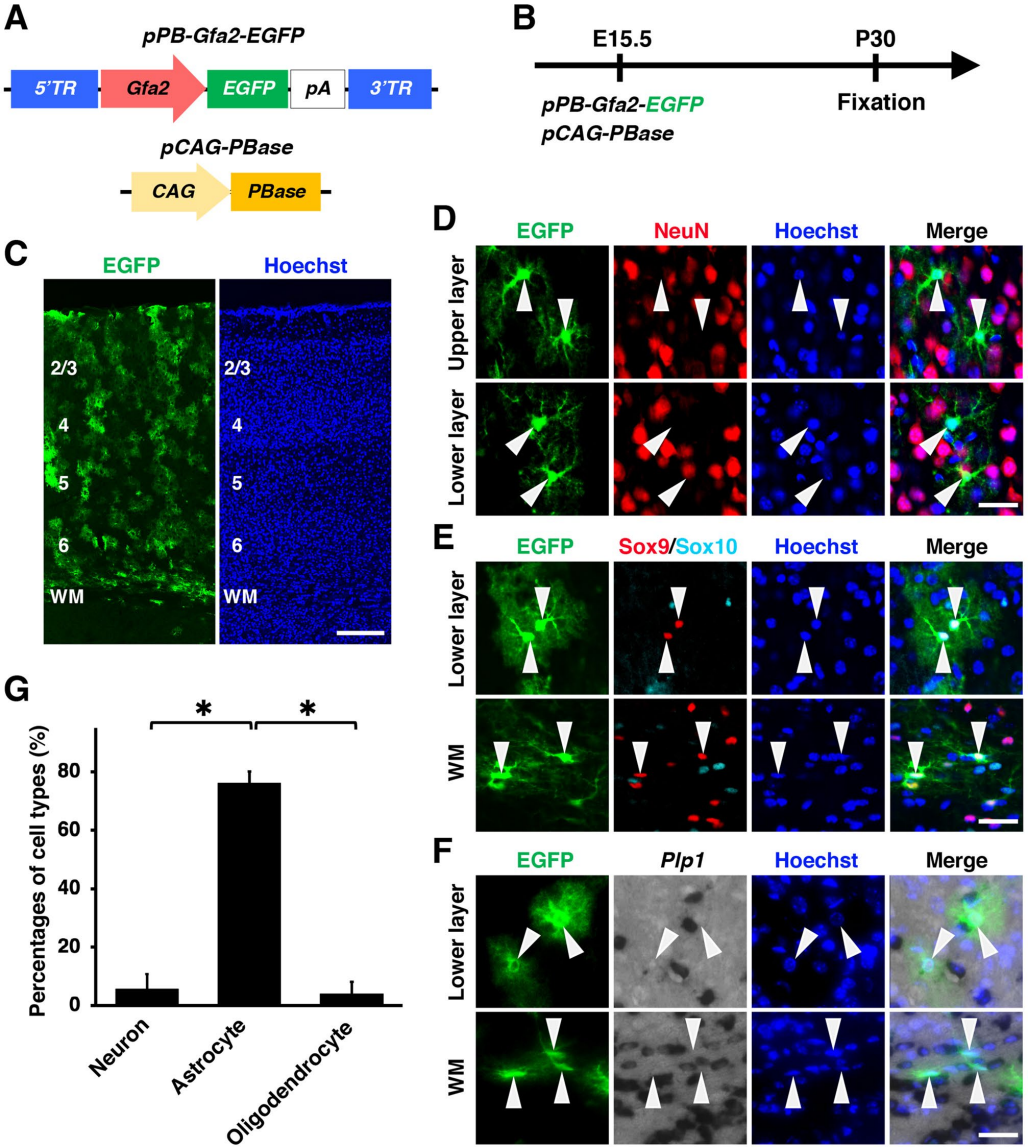
The *Gfa2* promoter induced transgene expression in astrocytes specifically in the mouse cerebrum. (**A**) Schematics of *pPB-Gfa2-EGFP* and *pCAG-PBase* plasmids. *pPB-Gfa2-EGFP* drives EGFP expression under the *Gfa2* promoter. (**B**) Experimental procedure. *pPB-Gfa2-EGFP* and *pCAG-PBase* were co-electroporated into the mouse cerebrum at E15.5, and coronal sections were prepared at P30. (**C**) Coronal sections of the electroporated cerebrum. Many EGFP-positive cells were distributed throughout the gray matter and the white matter. (**D**–**F**) Immunohistochemistry for NeuN (**D**), Sox9 and Sox10 (**E**), and in situ hybridization for *Plp1* (**F**). Most EGFP-positive cells expressed Sox9 and were negative for NeuN, Sox10 and *Plp1* (arrowheads). (**G**) The percentages of EGFP-positive cells which were also NeuN-positive (neuron), Sox9-positive/Sox10-negative (astrocyte) or *Plp1*-positive (oligodendrocyte). The *Gfa2* promoter drove EGFP expression in astrocytes selectively when combined with the *piggyBac* system and in utero electroporation. Unpaired Student’s *t*-test, *P < 0.0005. Error bars represent mean ± SD. The statistical analyses were performed using Microsoft Excel ver. 16.43. Scale bars: 200 μm (**C**), 30 μm (**D–F**). TR, terminal region; WM, white matter. Numbers indicate layers in the cerebral cortex.

We first electroporated *pPB-Gfa2-EGFP* and *pCAG-PBase* at E15.5 and prepared coronal sections of the cerebral cortex at P30 (Fig. 2A, B). Many EGFP-positive cells were distributed throughout the gray matter and the white matter (Fig. 2C) and exhibited morphological features of astrocytes (Fig. 2D–F, arrowheads). Immunohistochemistry and in situ hybridization revealed that most EGFP-positive cells expressed Sox9 and were negative for NeuN, Sox10 and *Plp1* (Fig. 2D–F, arrowheads), suggesting that most EGFP-positive cells were astrocytes. Our quantification gave results consistent with our observations (neuron, 5.6 ± 4.2%; astrocyte, 76.1 ± 7.6%; oligodendrocyte, 3.9 ± 2.4%) (Fig. 2G, Supplementary Fig. 1). These results indicate that EGFP-positive cells produced by *pPB-Gfa2-EGFP* and in utero electroporation are mostly astrocytes.

**Figure 3:**
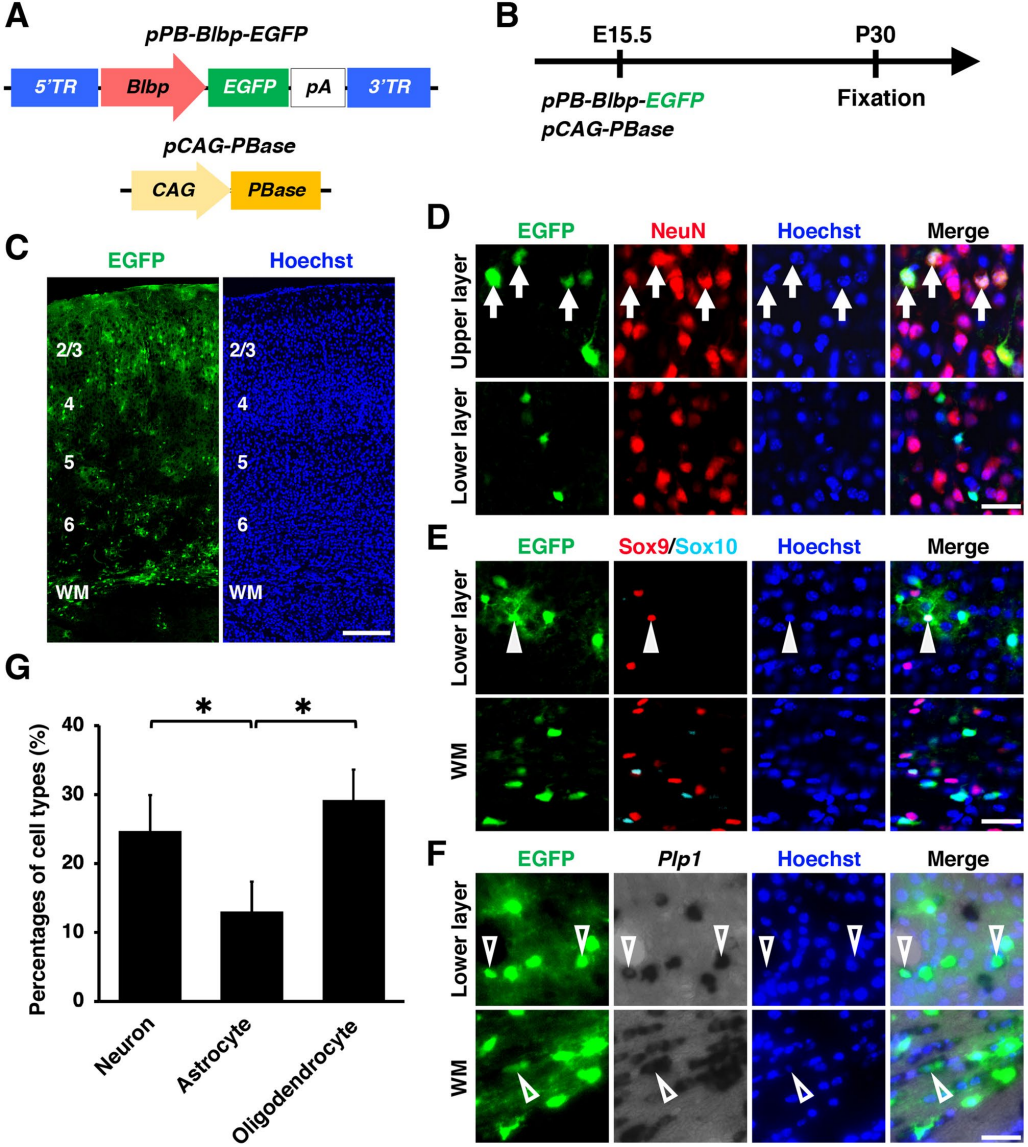
A *Blbp* promoter did not induce astrocyte-specific transgene expression when used with the *piggyBac* system and in utero electroporation. (**A**) Schematics of *pPB-Blbp-EGFP* and *pCAG-PBase* plasmids. *pPB-Blbp-EGFP* drives EGFP expression under a *Blbp* promoter. (**B**) Experimental procedure. *pPB-Blbp-EGFP* and *pCAG-PBase* were co-electroporated into the mouse cerebrum at E15.5, and coronal sections were prepared at P30. (**C**) Coronal sections of the electroporated cerebrum. Many EGFP-positive cells were distributed throughout the gray matter and the white matter. (**D**–**G**) Immunohistochemistry for NeuN (D), Sox9 and Sox10 (**E**), and in situ hybridization for *Plp1* (**F**). Although EGFP-positive cells contained Sox9-positive/Sox10-negative astrocytes (arrowheads), they also contained NeuN-positive neurons (arrows) and *Plp1*-positive oligodendrocytes (open arrowheads). (**G**) The percentages of EGFP-positive cells which were also NeuN-positive (neuron), Sox9-positive/Sox10-negative (astrocyte) or *Plp1*-positive (oligodendrocyte). This *Blbp* promoter drove gene expression not only in astrocytes but also in neurons and oligodendrocytes. Unpaired Student’s *t*-test, *P < 0.05. Error bars represent mean ± SD. The statistical analyses were performed using Microsoft Excel ver. 16.43. Scale bars: 200 μm (**C**), 30 μm (**D**–**F**). TR, terminal region; WM, white matter. Numbers indicate layers in the cerebral cortex.

We next electroporated *pPB-Blbp-EGFP* and *pCAG-PBase* (Fig. 3A, B). As in the case of *pPB-Gfa2-EGFP*, *pPB-Blbp-EGFP* produced many EGFP-positive cells throughout the gray matter and the white matter (Fig. 3C). However, although the EGFP-positive cells contained Sox9-positive/Sox10-negative astrocytes (Fig. 3E, arrowheads), many EGFP-positive cells were found to express NeuN (Fig. 3D, arrows) or *Plp1* (Fig. 3F, open arrowheads), suggesting that the EGFP-positive cells contained neurons and oligodendrocytes in the *pPB-Blbp-EGFP*-transfected brain. Our quantification showed that 25%, 13% and 29% of EGFP-positive cells were neurons, astrocytes and oligodendrocytes, respectively (neuron, 24.7 ± 5.2%; astrocyte, 13.0 ± 4.3%; oligodendrocyte, 29.3 ± 4.3%) (Fig. 3G), suggesting that the *Blbp* promoter drives gene expression not only in astrocytes but also in neurons and oligodendrocytes when it is used with the *piggyBac* system and in utero electroporation. Thus, these findings indicate that the *Gfa2* promoter rather than the *Blbp* promoter is suitable for selective gene expression in astrocytes in the mouse cerebral cortex using the *piggyBac* system and in utero electroporation.

A previous study revealed that the internal domain sequence of the 3′-terminal region (3′TR) itself of *piggyBac* may have the ability to induce rightward gene expression^34^. Therefore, it seemed possible that the low specificity of *pPB-Blbp-EGFP* resulted from gene expression driven by the 3′TR. Similarly, although most of the EGFP-positive cells induced by *pPB-Gfa2-EGFP* were astrocytes, a small number of neurons and oligodendrocytes were also labeled with EGFP. The 3′TR of *pPB-Gfa2-EGFP* could be responsible for the EGFP expression in neurons and oligodendrocytes, and the specificity of *pPB-Gfa2-EGFP* could be improved by removing the effects of the 3′TR. To test this possibility, because the 3′TR was reported to induce rightward gene expression, we inverted the directions of the *Blbp-EGFP* and the *Gfa2-EGFP* cassettes in *pPB* (Supplementary Figs. 2A, 3A). We performed in utero electroporation at E15.5 and prepared coronal sections at P30 (Supplementary Figs. 2B, 2C, 3B, 3C). We then performed immunohistochemistry for NeuN and Sox9/Sox10 and in situ hybridization for *Plp1* (Supplementary Figs. 2D–F, 3D–F). We found that the inversion of the cassettes did not reduce the percentages of EGFP-positive neurons and oligodendrocytes (*pPB-inverted-Gfa2-EGFP*; neuron, 13.4 ± 4.1%; astrocyte, 73.3 ± 3.5%; oligodendrocyte, 5.5 ± 4.5%) (*pPB-inverted-Blbp-EGFP*; neuron, 28.2 ± 6.1%; astrocyte, 8.9 ± 1.0%; oligodendrocyte, 33.7 ± 9.0%) (Supplementary Figs. 2G, 3G). These results suggest that 3′TR-induced rightward gene expression is not responsible for the EGFP expression in neurons and oligodendrocytes induced by *pPB-Blbp-EGFP* and *pPB-Gfa2-EGFP*.

### Promoters suitable for oligodendrocyte-specific gene expression

**Figure 4:**
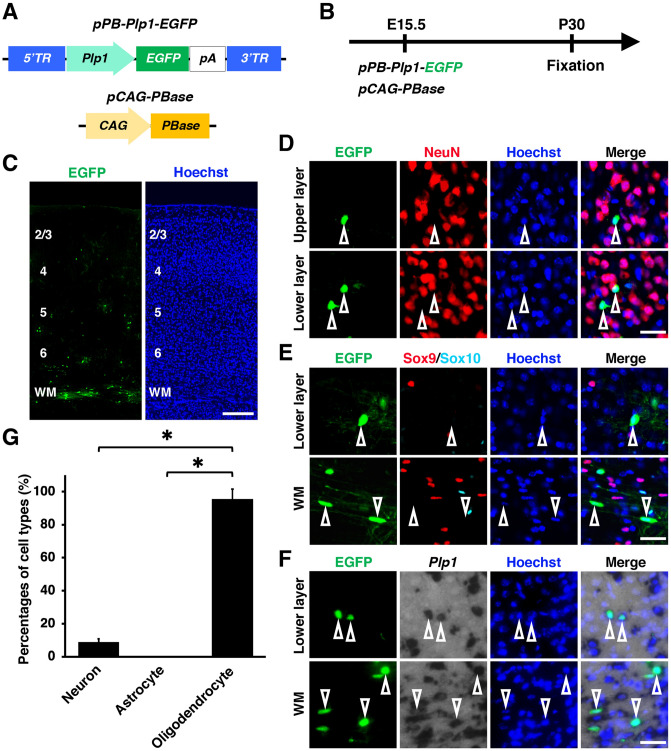
A *Plp1* promoter induced transgene expression in oligodendrocytes specifically in the mouse cerebrum. (**A**) Schematics of *pPB-Plp1-EGFP* and *pCAG-PBase* plasmids. *pPB-Plp1-EGFP* drives EGFP expression under a *Plp1* promoter. (**B**) Experimental procedure. *pPB-Plp1-EGFP* and *pCAG-PBase* were co-electroporated into the mouse cerebrum at E15.5, and coronal sections were prepared at P30. (**C**) Coronal sections of the electroporated cerebrum. Most EGFP-positive cells were distributed in the white matter. (**D–G**) Immunohistochemistry for NeuN (**D**), Sox9 and Sox10 (**E**), and in situ hybridization for *Plp1* (**F**). Most EGFP-positive cells were positive for *Plp1* and negative for NeuN and Sox9 (open arrowheads). (**G**) The percentages of EGFP-positive cells which are also NeuN-positive (neuron), Sox9-positive/Sox10-negative (astrocyte) or *Plp1*-positive (oligodendrocyte). This *Plp1* promoter drove gene expression in oligodendrocytes selectively when combined with the *piggyBac* system and in utero electroporation. Unpaired Student’s *t*-test, *P < 0.00005. Error bars represent mean ± SD. The statistical analyses were performed using Microsoft Excel ver. 16.43. Scale bars: 200 μm (**C**), 30 μm (**D-F**). TR, terminal region; WM, white matter. Numbers indicate layers in the cerebral cortex.

We next tried promoters which were reported to drive selective gene expression in oligodendrocytes using transgenic mice and retrovirus vectors. We utilized promoters derived from the *proteolipid protein 1* (*Plp1*) gene and the *myelin basic protein* (*Mbp*) gene^18,35^ (See Supplementary Table for details). We first electroporated *pPB-Plp1-EGFP* and *pCAG-PBase* at E15.5 and prepared brains at P30 (Fig. 4A, B). Interestingly, most EGFP-positive cells were distributed in the white matter, suggesting that they were mainly oligodendrocytes (Fig. 4C). Consistent with this idea, most EGFP-positive cells were positive for *Plp1* and negative for NeuN and Sox9 (Fig. 4D-F, open arrowheads). Our quantification showed that 9%, 0% and 95% of EGFP-positive cells were NeuN-positive neurons, Sox9-positive/Sox10-negative astrocytes and *Plp1*-positive oligodendrocytes, respectively (neuron, 8.9 ± 1.9%; astrocyte, 0%; oligodendrocyte, 95.4 ± 6.2%) (Fig. 4G). These results indicate that the *Plp1* promoter drives gene expression in oligodendrocytes selectively when combined with the *piggyBac* system and in utero electroporation.

**Figure 5:**
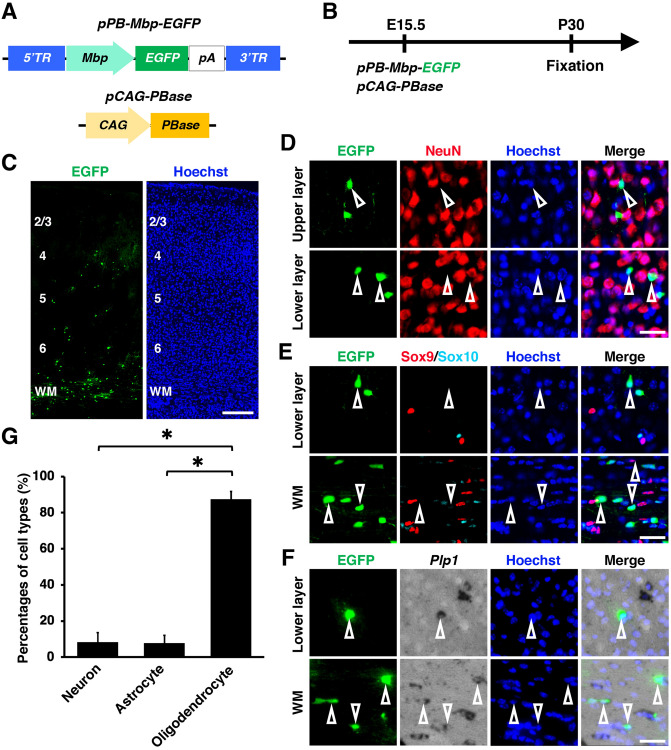
An *Mbp* promoter induced transgene expression into oligodendrocytes specifically in the mouse cerebrum. (**A**) Schematic of *pPB-Mbp-EGFP* and *pCAG-PBase* plasmid. *pPB-Mbp-EGFP* drives EGFP expression under an *Mbp* promoter. (**B**) Experimental procedure. *pPB-Mbp-EGFP* and *pCAG-PBase* were co-electroporated into the mouse cerebrum at E15.5, and coronal sections were prepared at P30. (**C**) Coronal sections of the electroporated cerebrum. Most EGFP-positive cells were accumulated in the white matter. (**D–G**) Immunohistochemistry for NeuN (**D**), Sox9 and Sox10 (**E**), and in situ hybridization for *Plp1* (**F**). Most EGFP-positive cells were positive for *Plp1* and negative for NeuN and Sox9 (open arrowheads). (**G**) The percentages of EGFP-positive cells which were also NeuN-positive (neuron), Sox9-positive/Sox10-negative (astrocyte) or *Plp1*-positive (oligodendrocyte). This *Mbp* promoter drove gene expression in oligodendrocytes selectively when combined with the *piggyBac* system and in utero electroporation. Unpaired Student’s *t*-test, *P < 0.00005. Error bars represent mean ± SD. The statistical analyses were performed using Microsoft Excel ver. 16.43. Scale bars: 200 μm (**C**), 30 μm (**D-F**). TR, terminal region; WM, white matter. Numbers indicate layers in the cerebral cortex.

We next electroporated *pPB-Mbp-EGFP* and *pCAG-PBase* (Fig. 5A, B). As in the case of *pPB-Plp1-EGFP*, EGFP-positive cells were accumulated in the white matter (Fig. 5C). Most EGFP-positive cells were positive for *Plp1* and negative for NeuN and Sox9 (Fig. 5D-F, open arrowheads). These results suggest that EGFP-positive cells are mainly oligodendrocytes. Consistent with this observation, our quantification demonstrated that almost 90% of EGFP-positive cells were *Plp1*-positive oligodendrocytes (neuron, 8.3 ± 5.0%; astrocyte, 7.7 ± 3.9%; oligodendrocyte, 87.5 ± 4.2%) (Fig. 5G). These results indicate that both the *Plp1* promoter and the *Mbp* promoter in the *piggyBac* donor plasmid are useful for oligodendrocyte-selective transgene expression in the cerebral cortex using in utero electroporation.

### Visualization of neuron-astrocyte and axon-oligodendrocyte interactions

**Figure 6:**
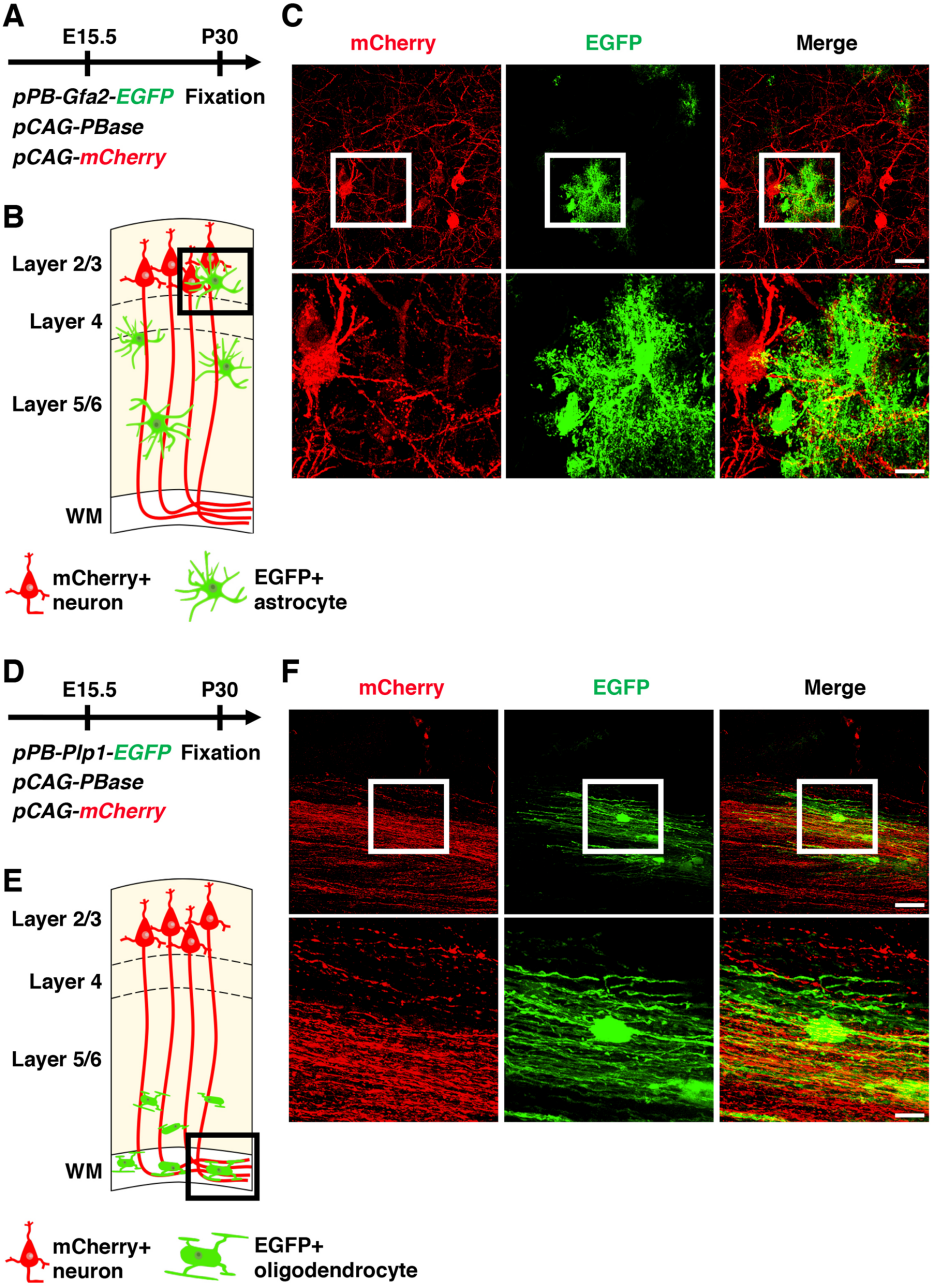
Visualization of neuron-astrocyte and axon-oligodendrocyte interactions. (**A**) Experimental procedure. *pPB-Gfa2-EGFP*, *pCAG-PBase* and *pCAG-mCherry* were co-electroporated into the mouse cerebrum at E15.5. Coronal sections were prepared at P30 and were stained with anti-RFP and anti-GFP antibodies. (**B**) Experimental design. Layer 2/3 neurons and astrocytes were labeled with mCherry and EGFP, respectively. Relationships between astrocytes and layer 2/3 neurons can be visualized (box). (**C**) Confocal images corresponding to the boxed area in (**B**). Magnified images of the areas within the boxes in the upper panels are shown in the lower panels. (**D**) Experimental procedure. *pPB-Plp1-EGFP*, *pCAG-PBase* and *pCAG-mCherry* were co-electroporated into the mouse cerebrum at E15.5. Coronal sections were prepared at P30 and were stained with anti-RFP and anti-GFP antibodies. (**E**) Experimental design. Layer 2/3 neurons and oligodendrocytes were labeled with mCherry and EGFP, respectively. The relationship between oligodendrocytes and axons of layer 2/3 neurons can be visualized (box). (**F**) Confocal images corresponding sto the boxed area in (**E**). Magnified images within the boxes of upper panels are shown in lower panels. Scale bars: 30 μm (**C** and **F**, upper panels), 10 μm (**C** and **F**, lower panels).

Close cooperation among neurons, astrocytes and oligodendrocytes is crucial for executing brain functions, and it would therefore be valuable to visualize interactions among neurons, astrocytes and oligodendrocytes in the brain. To label neurons and astrocytes with different colors, we introduced *pCAG-mCherry*, *pPB-Gfa2-EGFP* and *pCAG-PBase* using in utero electroporation at E15.5 (Fig. 6A). As described above, mCherry was expressed in layer 2/3 neurons, whereas EGFP was introduced into astrocytes selectively (Fig. 6B). Higher magnification confocal images successfully visualized the three-dimensional relationship between astrocytes and layer 2/3 neurons (Fig. 6C, Supplementary Movie 3).

We also labeled axons and oligodendrocytes with different colors. We introduced *pCAG-mCherry*, *pPB-Plp1-EGFP* and *pCAG-PBase* using in utero electroporation at E15.5 (Fig. 6D). mCherry was expressed in layer 2/3 neurons and their axons, and EGFP labeled oligodendrocytes (Fig. 6E). Higher magnification confocal images of the white matter clearly demonstrated oligodendrocytes sending their protrusions to neighboring axons (Fig. 6F, Supplementary Movie 4). These findings indicate that the combination of appropriate cell type-specific promoters, the *piggyBac* system and in utero electroporation is a powerful tool for the investigation of cell–cell interactions in the brain.

### EGFP expression in the germinal zones of the cerebral cortex

We also examined whether these plasmids induce transgene expression in neural progenitors. This seems important because plasmid concentrations should be higher in neural progenitors when introduced using in utero electroporation, and higher concentrations of plasmids may lead to promiscuous transgene expression. We electroporated *piggyBac* donor plasmids, *pCAG-PBase* and *pCAG-mCherry* at E15.5 and examined the EGFP expression pattern at E17.5 (Supplementary Fig. 4A). Strong mCherry signals in the germinal zones indicate high transfection efficiencies (Supplementary Fig. 4B). Interestingly, although *pPB-CAG-EGFP* induced strong EGFP expression in Pax6- and Tbr2-positive neural progenitors (Supplementary Fig. 4C, D), we did not see EGFP signals driven by *pPB-Plp1-EGFP* or *pPB-Mbp-EGFP* (Supplementary Fig. 4C, D). Weak EGFP signals were induced in neural progenitors by *pPB-Gfa2-EGFP* and *pPB-Blbp-EGFP* (Supplementary Fig. 4C, D, E, arrowheads). These expression patterns are consistent with those of endogenous *Plp1*, *Mbp*, Gfap and Blbp^36–39^. These results suggest that the promoters used in this study introduced by in utero electroporation recapitulate the expression patterns of endogenous genes in the germinal zones of the mouse cerebral cortex.

## Discussion

Here we have demonstrated that the *Gfa2* promoter is suitable for astrocyte-selective gene expression and that the *Plp1* and *Mbp* promoters used here are useful for selective gene expression in oligodendrocytes in the mouse cerebral cortex when combined with the *piggyBac* system and in utero electroporation. Furthermore, co-transfection of *pCAG-mCherry* and *pCAG-PBase* plus either *pPB-Gfa2-EGFP* or *pPB-Plp1-EGFP* enabled rapid examination of neuron-astrocyte or neuron-oligodendrocyte interactions, respectively. Our study provides a fundamental basis for specific transgene expression in astrocytes and/or oligodendrocytes in the mouse cerebral cortex.

In this study, we used four promoters which were reported to induce selective gene expression in astrocytes or oligodendrocytes in transgenic mice and retrovirus vectors^16–18,35^. Interestingly, although three out of four promoters resulted in the expected selective gene expression, the specificity of the *Blbp* promoter was not adequate when used in our system (Fig. 3). A possible reason for this is that a large copy number of the plasmid containing the *Blbp* promoter was integrated into the genomic DNA. It seems plausible that a large copy number of promoter regions leads to insufficient specificity of transgene expression. These results suggest that the specificities of promoters should be confirmed in advance when used with the *piggyBac* system and in utero electroporation, even if the promoters have been shown to be specific in transgenic mice. In addition, although the specificities of the promoters were examined in the mouse cerebral cortex at P30 in this study, their specificities could be different depending on age, location in the brain, animal species and plasmid dosage. Therefore, the specificities of the promoters should be re-validated in advance when new experimental conditions are used. Fortunately, because our system uses electroporation, confirming the specificities of promoters does not take a long time.

EGFP-positive oligodendrocytes induced by *pPB-Plp1-EGFP* and *pPB-Mbp-EGFP* were preferentially located in the white matter (Figs. 4C, 5C), whereas previous studies demonstrated that oligodendrocytes are distributed in both the gray matter and the white matter^38,40,41^. There are several possible reasons for this discrepancy. First, it seems possible that the *Plp1* promoter and the *Mbp* promoter used in this study did not induce transgene expression in all oligodendrocytes but instead induced transgene expression in a subset of oligodendrocytes which were located in the white matter. The second possibility is that these promoters induced transgene expression in all oligodendrocytes, but the transgene expression levels were not high enough in oligodendrocytes in the gray matter to allow for their visualization, and therefore, only oligodendrocytes with high EGFP expression in the white matter were visualized. Consistent with this possibility, a previous study showed higher Plp1 and Mbp expression in the white matter than in the gray matter^42^. It would be important to investigate whether all target cells can be labeled with the promoters used in this study.

Compared with other genetic manipulation techniques, our system has the following advantages for astrocyte- or oligodendrocyte-specific gene expression. First, specific transgene expression can be achieved rapidly and easily. Although various transgenic mice and knockin mice have been widely used to introduce transgenes in astrocytes or oligodendrocytes selectively, the processes for generating new transgenic mice and knockin mice are arduous^13–16^. In addition, although conditional gene targeting can be induced by crossing floxed mice and Cre-expressing mice under the control of astrocyte- or oligodendrocyte-specific promoters, breeding and mating of these mouse lines are relatively time-consuming^43–46^. Our findings demonstrated that combining glial cell-specific promoters, the *piggyBac* system and in utero electroporation allow astrocyte- or oligodendrocyte-specific genetic manipulation within a few weeks. Furthermore, by integrating Cre recombinase into our system and using floxed mice, astrocyte- or oligodendrocyte-specific gene knockout should be obtained rapidly.

Second, multiple characteristics of astrocytes or oligodendrocytes can be investigated simultaneously using our system. This is because multiple kinds of plasmids can be introduced into the same cells using in utero electroporation^47^. Furthermore, our system allows the introduction of distinct transgenes into neurons, astrocytes and oligodendrocytes differentially, as shown in Fig. 6. Therefore, our system would be useful for investigating the interactions between neurons and glial cells. Simultaneous analyses of morphology, calcium signals, synapse-related proteins and intracellular signals would be intriguing^47^.

Third, our system might be applicable to mammals other than mice. The brain has greatly developed during evolution, and carnivores and primates including humans have more developed brains than rodents such as mice. It is therefore important to investigate the brains of carnivores and primates using genetic techniques. Because we recently succeeded in establishing an in utero electroporation procedure for gyrencephalic carnivore ferrets^21,26,48^, our system described in this study might be useful for investigating various features of astrocytes and oligodendrocytes in ferrets. Previous studies have reported that the morphology of astrocytes has markedly changed during evolution^49,50^. It would be intriguing to investigate the similarities and differences of astrocytes in mice and ferrets using our system.

## Methods

### Animals

ICR mice were purchased from SLC, Japan. The day of conception and that of birth were counted as embryonic day 0 (E0) and postnatal day 0 (P0), respectively. All procedures were approved by the Animal Care Committee of Kanazawa University and were performed in accordance with relevant guidelines and regulations.

### In utero electroporation

In utero electroporation was performed as described previously^51,52^. Pregnant ICR mice were anesthetized, and the uterine horns were exposed. Approximately 1–3 μl of DNA solution was injected into the lateral ventricle of embryos using a pulled glass micropipette. Each embryo within the uterus was placed between tweezer-type electrodes (CUY650P3, NEPA Gene, Japan), and square electric pulses (45 V, 50 ms) were passed 5 times at 1 Hz using an electroporator (ECM830, Harvard Apparatus). Care was taken to quickly place embryos back into the abdominal cavity to avoid temperature loss. The wall and skin of the abdominal cavity were sutured.

### Plasmids

*pCAG-EGFP* and *pCAG-mCherry* were described previously^51,52^. *pPB-CAG-EiP* was kindly provided by Dr. Akitsu Hotta^53^. *pCAG-PBase* was kindly provided by Dr. Knut Woltjen^54^.

For the construction of *pPB-CAG-MCS-EGFP*, a *CAG-EGFP* DNA fragment was amplified from *pCAG-EGFP*. Using the In-Fusion method, the amplified fragment was inserted into *pPB-CAG-EiP* digested with XhoI and NheI, yielding *pPB-CAG-EGFP*. Oligonucleotides for a multiple cloning site (MCS) and an *EGFP* DNA fragment amplified from *pPB-CAG-EGFP* were inserted into *pPB-CAG-EGFP* digested with EcoRI to yield *pPB-CAG-MCS-EGFP* using the In-Fusion method.

For the construction of *pPB-Gfa2-EGFP*, *pPB-Blbp-EGFP*, *pPB-Mbp-EGFP* and *pPB-Plp1-EGFP*, *pPB-CAG-MCS-EGFP* was digested with SalI and then self-ligated to remove the CAG promoter, which yielded *pPB-MCS-EGFP*. The *Gfa2* promoter of *pGfa2-LacZ* (provided by Dr. Michael Brenner)^17^, the *Blbp* promoter of *pCAT3-Blbp-EGFP* (provided by Dr. Eva S. Anton)^16^, the *Mbp* promoter of *Mbp-pIP200* (provided by the late Dr. Kazuhiro Ikenaka)^35^ and the *Plp1* promoter of *Plp1-SV40* (provided by the late Dr. Kazuhiro Ikenaka)^18^ were isolated with BglII-NotI, XhoI-ApaI, HindIII and ApaI-AscI, respectively, and inserted into the corresponding MCS sites of the *pPB-MCS-EGFP* plasmid. Detailed information about the promoters used in this study is written in the Supplementary Table.

For the construction of *pPB-inverted-Gfa2-EGFP* and *pPB-inverted-Blbp-EGFP*, in order to isolate the *Gfa2-EGFP-polyA* and the *Blbp-EGFP-polyA* cassettes, *pPB-Gfa2-EGFP* and *pPB-Blbp-EGFP* were digested with PacI, blunted and further digested with SalI. *pPB-CAG-EiP* was digested with NheI, blunted and further digested with XhoI to remove the *CAG-EiP* fragment, yielding the *pPB* vector. Then, the *Gfa2-EGFP-polyA* and the *Blbp-EGFP-polyA* cassettes were inserted into the *pPB* vector.

### Preparation of tissue sections

Preparation of tissue sections was performed as described previously^55,56^. Mice at P30 were deeply anesthetized and transcardially perfused with 4% paraformaldehyde (PFA)/PBS. Brains were dissected and post-fixed with overnight immersion in 4% PFA/PBS, cryoprotected with 2-day immersion in 30% sucrose/PBS, and embedded in OCT compound. Coronal sections of 14 μm and 50 μm thickness were made using a cryostat.

### Immunohistochemistry

Immunohistochemistry was performed as described previously slight modifications^57,58^. Coronal sections were permeabilized with 0.3% Triton X-100/PBS and incubated overnight with primary antibodies in 2% bovine serum albumin (BSA)/PBS, then washed with 0.3% Triton X-100/PBS three times for 5 min each. The sections were incubated with secondary antibodies and Hoechst 33342 in 2% BSA/PBS for 2 h, and then washed with 0.3% Triton X-100/PBS three times for 5 min each. The sections were mounted on slides with Mowiol (Sigma-Aldrich). For immunohistochemistry using anti-Sox9 and anti-Sox10 antibodies, sections were incubated in antigen retrieval solution (10 mM citrate, pH 6.0) for 30 min at 70℃ before the incubation with primary antibodies. Antibodies used here were as follows: anti-NeuN antibody (Cell Signaling Technology), anti-Sox9 antibody (R&D systems), anti-Sox10 antibody (Santa Cruz Biotechnology), anti-Pax6 antibody (Millipore), anti-Tbr2 antibody (R&D systems), secondary antibodies conjugated with Alexa Fluor 647 (Molecular Probe), and secondary antibodies conjugated with Cy3 (Jackson ImmunoResearch).

### In situ hybridization

In situ hybridization using digoxigenin-labeled RNA probes was performed as described previously^28,59,60^. Briefly, sections of 14 μm thickness were incubated overnight with digoxigenin-labeled RNA probes in hybridization buffer (50% formamide, 5 × SSC, 5 × Denhardt’s solution, 0.3 mg/ml yeast RNA, 0.1 mg/ml herring sperm DNA, and 1 mM dithiothreitol). The sections were then incubated with an alkaline phosphatase-conjugated anti-digoxigenin antibody (Roche) and were visualized using NBT/BCIP as substrates. In addition, the sections were then subjected to Hoechst 33342 staining. The RNA probe for mouse *Plp1* used here was described previously^28^.

### Microscopy

Epifluorescence microscopy was performed with a BZ-X710 microscope (KEYENCE). Confocal microscopy was performed with a Nikon Eclipse Ti2 confocal microscope (Nikon) equipped with the Perfect Focus System, an Andor Dragonfly spinning-disk unit and an Andor EMCCD camera (Oxford Instruments). Three-dimensional images and movies of glial cells were acquired using Imaris software ver. 9.1.2 (Oxford Instruments).

### Quantification and statistical analyses

For quantification, coronal sections containing abundant EGFP signals were used. Coronal sections were subjected to immunohistochemistry for NeuN, double-immunohistochemistry for Sox9 and Sox10, or in situ hybridization for *Plp1*. The sections were also stained with Hoechst 33342. EGFP-positive regions in the mediolateral cerebrum were analyzed. Images captured using the sectioning module of a BZ-X710 microscope (KEYENCE) were used for quantification. Background signals were removed by subtracting the average signal intensities of EGFP-negative regions. The numbers of NeuN-positive, Sox9-positive/Sox10-negative, *Plp1*-positive cells or EGFP-positive cells were counted using the cell counter tool of ImageJ software ver. 2.0.0-rc-68/1.52 h.

For quantification of the percentages of EGFP-positive cells which were also NeuN-positive, Sox9-positive/Sox10-negative, or *Plp1*-positive, at least three brains for each condition were used.

Values in graphs represent mean ± SD. Unpaired two-tailed Student’s *t*-test was used to determine the statistical significance. *P* < 0.05 was considered as statistically significant.

## Supplementary Information

Supplementary Video 1.

Supplementary Video 2.

Supplementary Video 3.

Supplementary Video 4.

Supplementary Information 1.

Supplementary Information 2.
