# Supplementary material for: Glial cell type-specific gene expression in the mouse cerebrum using the piggyBac system and in utero electroporation
Source: Sci Rep. 2021 Mar 1;11:4864. doi: 10.1038/s41598-021-84210-z (PMC7921133; doi:10.1038/s41598-021-84210-z)
Supplement: Supplementary file 5 — Supplementary Information 1. [file 41598_2021_84210_MOESM5_ESM.pdf]

Supplementary Materials

Glial cell type-specific gene expression in the mouse cerebrum using the *piggyBac* system and *in utero* electroporation

Toshihide Hamabe-Horiike, Kanji Kawasaki, Masataka Sakashita, Chihiro Ishizu, Tomokazu Yoshizaki, Shin-ichi Harada, Keiko Ogawa-Ochiai, Yohei Shinmyo and Hiroshi Kawasaki

Supplementary Figure 1
Supplementary Figure 2
Supplementary Figure 3
Supplementary Figure 4
Supplementary Table
Supplementary Movie 1
Supplementary Movie 2
Supplementary Movie 3
Supplementary Movie 4

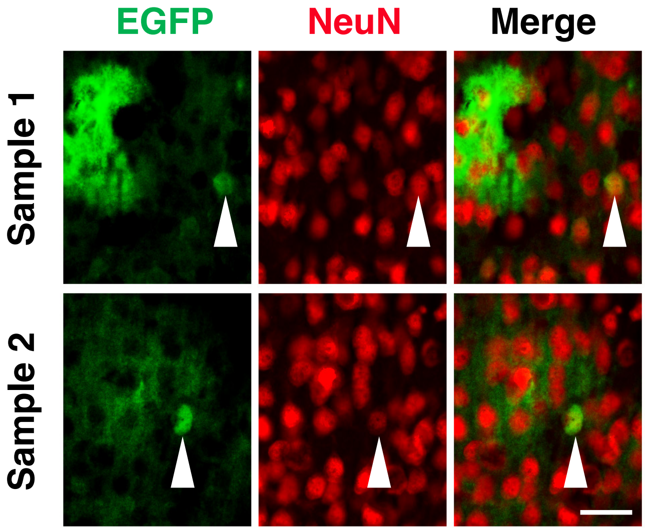

Supplementary Figure 1. EGFP-positive cells co-expressing NeuN in the *pPB-Gfa2-EGFP*-electroporated cortex.

pPB-Gfa2-EGFP and *pPB-CAG-PBase* were co-electroporated into the mouse cerebral cortex at E15.5, and coronal sections were prepared at P30. Immunohistochemistry for NeuN was performed. A few EGFP-positive cells co-expressed NeuN (arrowheads). Scale bar: 30 μm.

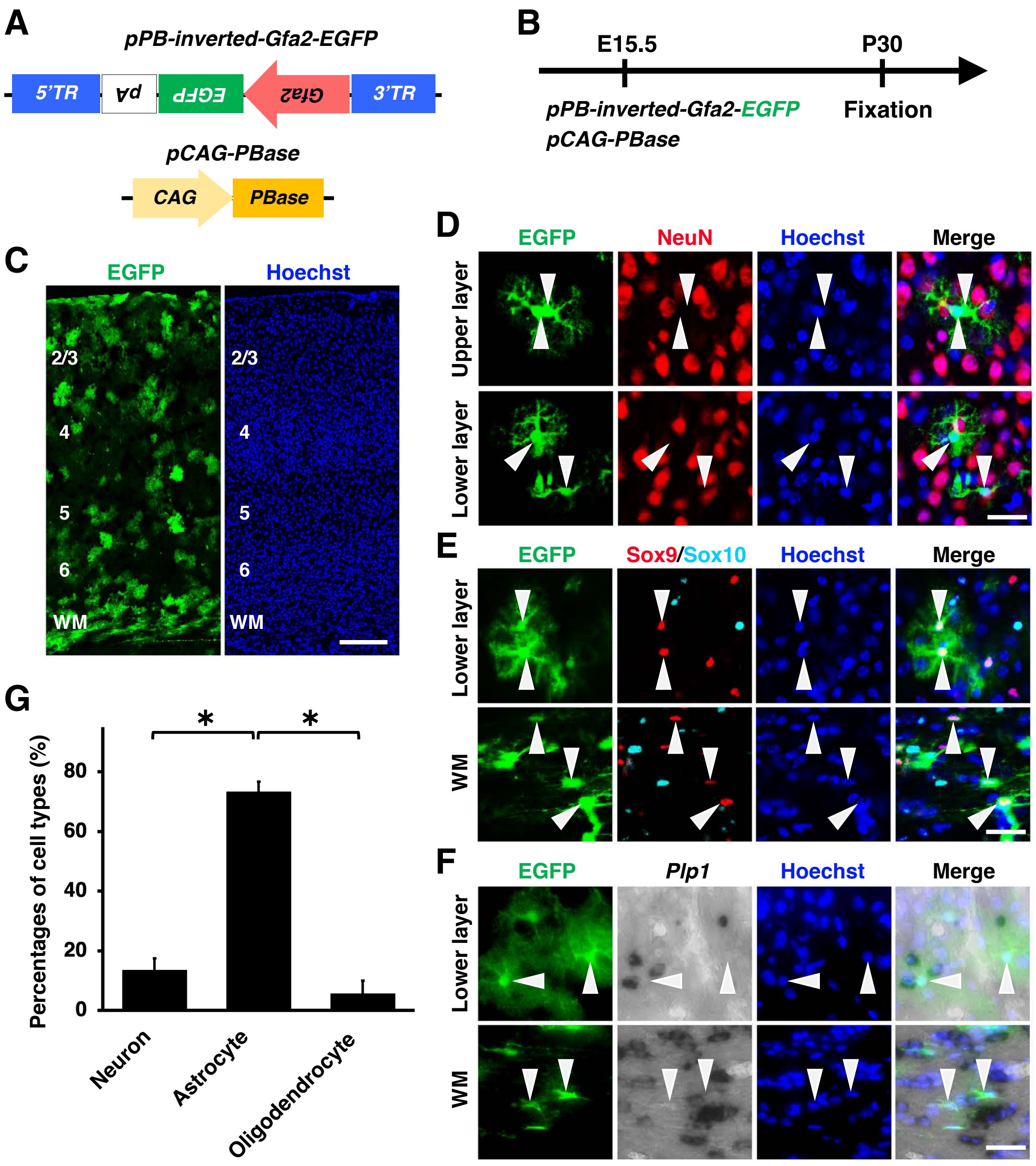

Supplementary Figure 2. EGFP expression induced by *pPB-inverted-Gfa2-EGFP*. **(A)** Schematics of *pPB-inverted-Gfa2-EGFP* and *pCAG-PBase* plasmids. **(B)** Experimental procedure. *pPB-inverted-Gfa2-EGFP* and *pCAG-PBase* were co-electroporated into the mouse cerebral cortex at E15.5, and coronal sections were prepared at P30. **(C)** Coronal sections of the electroporated cerebrum. Many EGFP-positive cells were distributed throughout the gray matter and the white matter. **(D-G)** Immunohistochemistry for NeuN (**D**), Sox9 and Sox10 (**E**), and *in situ* hybridization for *Plp1* (**F**). Many EGFP-positive cells expressed Sox9 and were negative for NeuN, Sox10

and *Plp1* (arrowheads). **(G)** The percentages of EGFP-positive cells which were also NeuN-positive (neuron), Sox9-positive/Sox10-negative (astrocyte) or *Plp1*-positive (oligodendrocyte). Unpaired Student's *t*-test, $*p < 0.0005$. Error bars represent mean ± SD. The statistical analyses were performed using Microsoft Excel ver. 16.43. Scale bars: 200 μm (**C**), 30 μm (**D-F**). WM, white matter. Numbers indicate layers in the cerebral cortex.

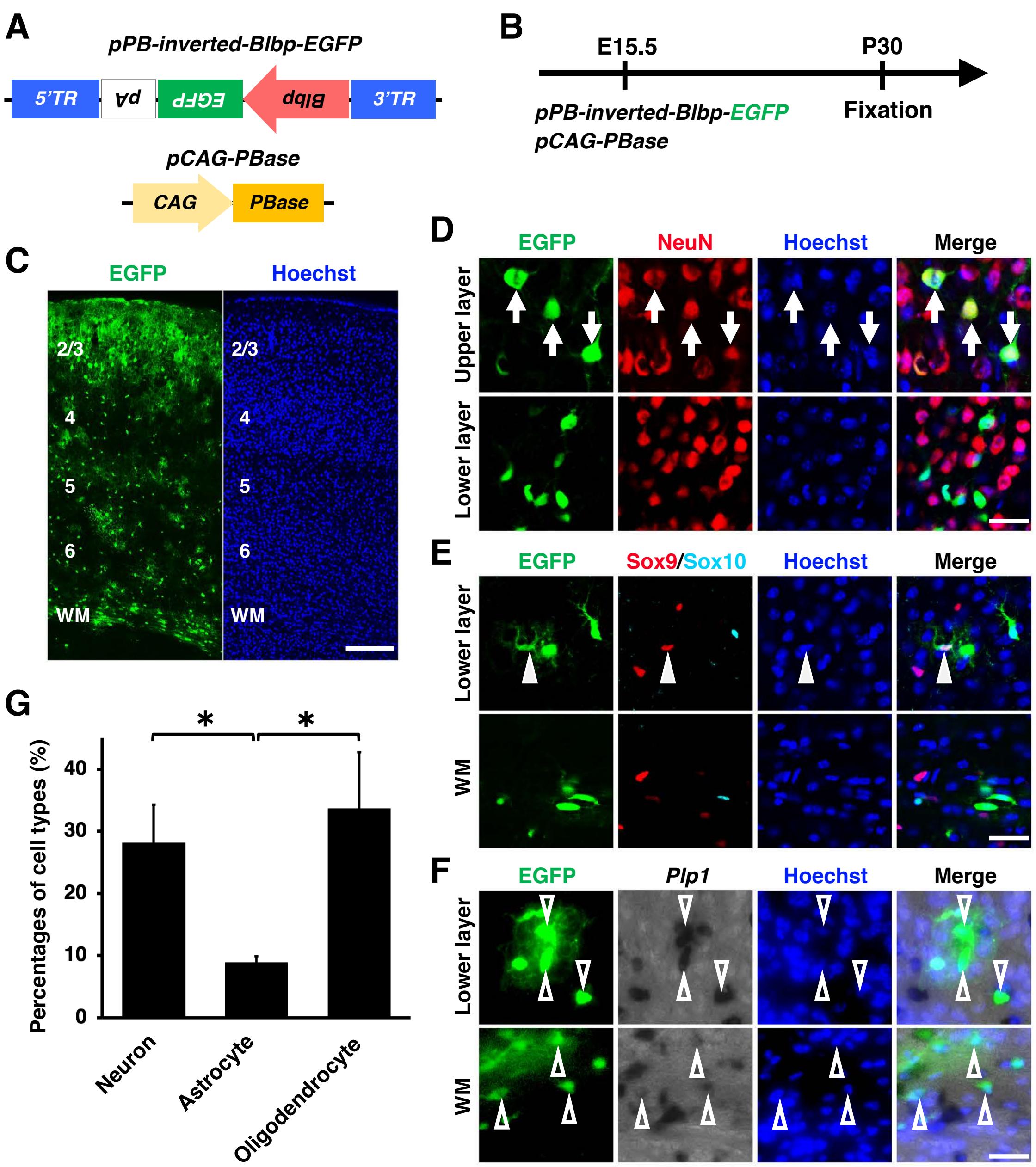

Supplementary Figure 3. EGFP expression induced by *pPB-inverted-Blbp-EGFP*. **(A)** Schematics of *pPB-inverted-Blbp-EGFP* and *pCAG-PBase* plasmids. **(B)** Experimental procedure. *pPB-inverted-Blbp-EGFP* and *pCAG-PBase* were co-electroporated into the mouse cerebral cortex at E15.5, and coronal sections were prepared at P30. **(C)** Coronal sections of the electroporated cerebrum. Many EGFP-

positive cells were distributed throughout the gray matter and the white matter. **(D-G)** Immunohistochemistry for NeuN (**D**), Sox9 and Sox10 (**E**), and *in situ* hybridization for *Plp1* (**F**). Although EGFP-positive cells contained Sox9-positive/Sox10-negative astrocytes (arrowheads), they also contained NeuN-positive neurons (arrows) and *Plp1*-positive oligodendrocytes (open arrowheads). **(G)** The percentages of EGFP-positive cells which were also NeuN-positive (neuron), Sox9-positive/Sox10-negative (astrocyte) or *Plp1*-positive (oligodendrocyte). Unpaired Student's *t*-test, $*p < 0.01$. Error bars represent mean ± SD. The statistical analyses were performed using Microsoft Excel ver. 16.43. Scale bars: 200 μm (**C**), 30 μm (**D-F**). WM, white matter. Numbers indicate layers in the cerebral cortex.

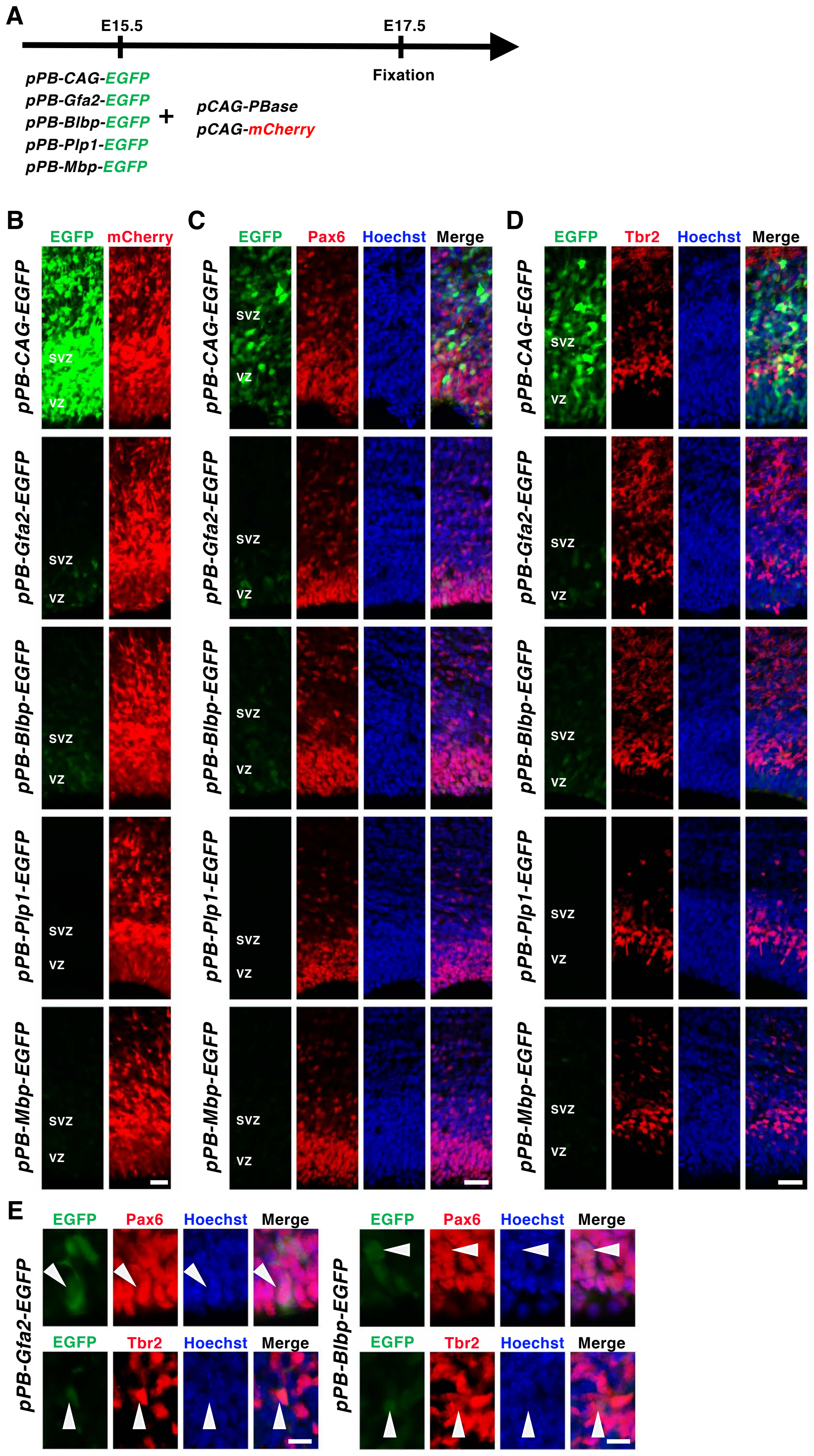
A
E15.5
E17.5
Fixation
pPB-CAG-EGFP
pPB-Gfa2-EGFP
pPB-Blbp-EGFP
pPB-Plp1-EGFP
pPB-Mbp-EGFP
+
pCAG-PBase
pCAG-mCherry
B
EGFP
mCherry
C
EGFP
Pax6
Hoechst
Merge
D
EGFP
Tbr2
Hoechst
Merge
SVZ
VZ
E
pPB-Gfa2-EGFP
pPB-Blbp-EGFP

Supplementary Figure 4. EGFP expression in neural progenitors in the cerebral cortex.

(A) Experimental procedure. *pCAG-mCherry* and *pCAG-PBase* plus either *pPB-CAG-EGFP*, *pPB-Gfa2-EGFP*, *pPB-Blbp-EGFP*, *pPB-Mbp-EGFP* or *pPB-Plp1-EGFP* were co-electroporated into the mouse cerebral cortex at E15.5. Coronal sections were prepared 2 days later, and Hoechst 33342 staining and immunostaining for Pax6 and Tbr2 were performed. (**B**) Lower magnification images of the germinal zone of the cerebral cortex. Note the abundant mCherry signals in all five samples, indicating high electroporation efficiencies in all five samples. (**C-E**) Immunohistochemistry for Pax6 and Tbr2. Lower magnification images (**C, D**) and higher magnification images (**E**) of the germinal zone are shown. Strong EGFP signals in the germinal zone were induced by *pPB-CAG-EGFP*. In contrast, no EGFP signals were observed in the germinal zones of *pPB-Plp1-EGFP*- and *pPB-Mbp-EGFP*-electroporated cortices. Weak EGFP signals were observed in Pax6-positive and Tbr2-positive neural progenitors in the germinal zones of *pPB-Gfa2-EGFP*- and *pPB-Blbp-EGFP*-electroporated cortices (**E**, arrowheads). VZ, ventricular zone; SVZ, subventricular zone. Scale bars: 30 μm (**B-D**), 10 μm (**E**).
