# Supplementary material for: Glial cell type-specific gene expression in the mouse cerebrum using the piggyBac system and in utero electroporation
Source: Sci Rep. 2021 Mar 1;11:4864. doi: 10.1038/s41598-021-84210-z (PMC7921133; doi:10.1038/s41598-021-84210-z)
Supplement: Supplementary file 6 — Supplementary Information 2. [file 41598_2021_84210_MOESM6_ESM.pdf]

Supplementary Table

Promoters used for astrocyte- or oligodendrocyte-specific gene expression

Gene	Accession No.	Length	Region	Reference
human GFAP	M67446	2.2 kb	-2163 to +47 bp relative to the transcriptional start site.<br>The initiating ATG at +15 bp was changed to TTG.	This study<br>Ref. 17
human GFAP	M67446	694 bp	From the 2.2 kb human *GFAP* promoter, 5' nucleotides -2163 to -1758 and an internal segment from -1255 to -133 were deleted.	Ref. 15
mouse Blbp	U04827	824 bp	-773 to +51 bp relative to the transcriptional start site.	This study<br>Ref. 16
mouse Plp1	AF003838<br>AL671887<br>X07215<br>X07216	11.3 kb	2410 bp of the 5'-flanking DNA, all of exon 1 and intron 1, and first 37 bp of exon 2.<br>The initiating ATG in exon 1 was changed to GAG.	This study<br>Ref. 18
mouse Plp1	AF003838<br>AL671887<br>X07215<br>X07216	11.3 kb	2410 bp of the 5'-flanking DNA, all of exon 1 and intron 1, and the first 24 bp of exon 2.<br>The initiating ATG in exon 1 was changed to GAG.	Ref. 14
mouse Mbp	M24410	346 bp	-256 to +90 bp relative to the transcriptional start site.	Ref. 13
mouse Mbp	M24410	1.3 kb	-1317 to +13 bp relative to the transcriptional start site.	This study<br>Ref. 35
